# Quantitative bone imaging biomarkers and joint space analysis of the articular Fossa in temporomandibular joint osteoarthritis using artificial intelligence models

**DOI:** 10.3389/fdmed.2022.1007011

**Published:** 2022-09-19

**Authors:** Tamara Mackie, Najla Al Turkestani, Jonas Bianchi, Tengfei Li, Antonio Ruellas, Marcela Gurgel, Erika Benavides, Fabiana Soki, Lucia Cevidanes

**Affiliations:** 1 Department of Orthodontics and Pediatric Dentistry, University of Michigan, Ann Arbor, MI, United States; 2 Department of Restorative and Aesthetic Dentistry, Faculty of Dentistry, King Abdulaziz University, Jeddah, Saudi Arabia; 3 Department of Orthodontics, University of the Pacific, Arthur Dugoni School of Dentistry, San Francisco, CA, United States; 4 Department of Radiology and Biomedical Research Imaging Center, University of North, Chapel Hill, NC, United States; 5 Department of Orthodontics, Federal University of Rio de Janeiro, Rio de Janeiro, Brazil; 6 Department of Periodontics and Oral Medicine, University of Michigan, Ann Arbor, MI, United States

**Keywords:** articular fossa, imaging biomarkers, hr-CBCT, joint space, temporomandibular osteoarthritis, artificial intelligence

## Abstract

Temporomandibular joint osteoarthritis (TMJ OA) is a disease with a multifactorial etiology, involving many pathophysiological processes, and requiring comprehensive assessments to characterize progressive cartilage degradation, subchondral bone remodeling, and chronic pain. This study aimed to integrate quantitative biomarkers of bone texture and morphometry of the articular fossa and joint space to advance the role of imaging phenotypes for diagnosis of Temporomandibular Joint Osteoarthritis (TMJ OA) in early to moderate stages by improving the performance of machine-learning algorithms to detect TMJ OA status. Ninety-two patients were prospectively enrolled (184 h-CBCT scans of the right and left mandibular condyles), divided into two groups: 46 control and 46 TMJ OA subjects. No significant difference in the articular fossa radiomic biomarkers was found between TMJ OA and control patients. The superior condyle-to-fossa distance (*p* < 0.05) was significantly smaller in diseased patients. The interaction effects of the articular fossa radiomic biomarkers enhanced the performance of machine-learning algorithms to detect TMJ OA status. The LightGBM model achieved an AUC 0.842 to diagnose the TMJ OA status with Headaches and Range of Mouth Opening Without Pain ranked as top features, and top interactions of VE-cadherin in Serum and Angiogenin in Saliva, TGF-*β*1 in Saliva and Headaches, Gender and Muscle Soreness, PA1 in Saliva and Range of Mouth Opening Without Pain, Lateral Condyle Grey Level Non-Uniformity and Lateral Fossa Short Run Emphasis, TGF-*β*1 in Serum and Lateral Fossa Trabeculae number, MMP3 in Serum and VEGF in Serum, Headaches and Lateral Fossa Trabecular spacing, Headaches and PA1 in Saliva, and Headaches and BDNF in Saliva. Our preliminary results indicate that condyle imaging features may be more important in regards to main effects, but the fossa imaging features may have a larger contribution in terms of interaction effects. More studies are needed to optimize and further enhance machine-learning algorithms to detect early markers of disease, improve prediction of disease progression and severity to ultimately better serve clinical decision support systems in the treatment of patients with TMJ OA.

## Introduction

The Diagnostic Criteria (DC) for TemporoMandibular Joint Disorders (TMD) have recently described the condition of TemporoMandibular Joint Osteoarthritis (TMJ OA) defined by Ahmad et al 2009 (1) as Degenerative Joint Disease (2). In this study, we use the 2009 term, TMJ OA, because this is a disease with a multifactorial etiology, involving many pathophysiological processes, and requires comprehensive assessments to characterize progressive cartilage degradation, subchondral bone remodeling, and chronic pain (3–5). TMJ OA – once thought to be a condition involving “wear and tear” over time – is now classified as a “low inflammatory arthritic condition” (6) and associated with inflammatory mediators that lead to proliferative and resorptive inflammatory response with overall destructive consequences on the structural components of the TMJ, such as its cartilage, bone, and synovium (7). The progression of TMJ-OA may be slow (8), and the initial stages may be subclinical until the disease process has advanced to chronical stages (9). The TMJ provides a unique model to study early bone changes in OA, as only a thin layer of fibrocartilage covers the articular bone surface in the TMJ condyle (10, 11). Numerous animal studies indicate that the bone microarchitecture (4, 5, 12) is an important factor in the OA pathogenesis initiation, preceding articular cartilage changes (12, 13), and should be investigated in human studies for early TMJ OA detection. As treatments to reverse the chronic damage of TMJ OA are for the most part unavailable and limited (14), it is clear that early diagnosis may provide the best opportunity to prevent extensive and permanent joint damage. Current diagnosis standard protocols recommended in the DC/TMD criteria (1, 2) are based on pre-existent condylar damage, such as subcortical cysts, surface erosions, osteophytes, or generalized sclerosis.

Radiomics is the conversion of digital medical images into mineable high-dimensional data (15) – it refers to the extraction and analysis of advanced quantitative imaging from medical images to diagnose and/or predict diseases. This process is motivated by the concept that biomedical images contain information that reflect underlying pathophysiology and that these relationships can be revealed *via* quantitative image analyses (15). With high-throughput computing, it is now possible to promptly obtain countless quantitative features from relatively new high resolution low radiation CBCT (hr-CBCT) (16), and new software applications, with a user-friendly interface, can now easily extract large amounts of quantitative features from hr-CBCT greyscale images (17, 18). A study conducted by Bianchi et al. using quantitative bone imaging biomarkers for diagnosis of TMJ OA from hr-CBCT scans of mandibular condyles showed differences in subchondral bone microstructure between control and TMJ OA groups, and that they provided an acceptable diagnostic performance for the diagnosis of TMJ-OA. This opens up the notion that these biomarkers could be clinically significant in recognizing early onset of TMJ OA and enabling early, conservative therapy (19).

This study seeks to investigate whether the inclusion of articular fossa data improves the performance of machine-learning algorithms to detect TMJ OA status. Dislocation of the mandibular condyle from the articular fossa (mimicking the absence of the condyle) results in the arrested development of the fossa (20). This suggests that normal fossa development depends on normal condyle development, and the fossa bony microstructure may show signs of TMJ OA comparable to the condyle. Literature on changes related to the articular fossa in patients with TMJ OA is limited to roof thickness and joint space narrowing (21–22). The present study aims specifically to evaluate whether the integration of condyle-to-fossa distances and quantitative bone texture and morphometry imaging biomarkers in the articular fossa improve the performance of machine-learning algorithms for the diagnosis of TMJ OA in early to moderate stages.

## Materials and methods

This study followed the STROBE guidelines for observational studies. This cross-sectional study was approved by the Institutional Review Board of the University of Michigan (HUM00113199). All patients signed an informed consent and agreed to participate.

### Study design and participants

The following inclusion criteria were applied for all patients: age between 21 and 70 years, no history of systemic disease, no history of TMJ trauma, surgery, or recent TMJ injections, no current pregnancy, and no congenital bone or cartilage disease. The control subjects were recruited by advertisements placed in the University Of Michigan School Of Dentistry and at The University of Michigan Dentistry Hospital; potential participants were first screened by telephone interview. The TMJ OA patients were recruited at their appointment with the TMD specialist from the University of Michigan. A total of 92 patients were selected, for a total 184 h-CBCT scans of the mandibular condyles. All subjects were clinically evaluated by the same TMD specialist using the Diagnostic Criteria for Temporomandibular Disorders (DC/TMD) (1–2) They were then divided into two groups: a control group (*n* = 46 patients, 46 condyles) and a TMJ OA group (*n* = 46 patients, 46 condyles). The inclusion criteria for control subjects were no history of clinical signs/symptoms of TMD. The inclusion criteria for the TMJ OA group were the presence of TMJ pain for less than 10 years, with clinical signs and symptoms evaluated using the DC/TMD: TMJ noise during movement or function in the last 30 days and crepitus detected during mandibular excursive movements. The radiographic CBCT interpretation was conducted by two oral and maxillofacial radiologists to confirm the presence of TMJ OA and was positive for at least one of the following: subchondral cyst, erosion, generalized sclerosis, and/or osteophytes (1). The exclusion criteria for the TMJ OA group were subjects with more than 10 years since the diagnosis of TMJ OA, or condyles with severe stages of bone destruction, subchondral cyst, erosion, generalized sclerosis, and/or osteophytes. The subjects were age and sex matched, with a mean 36 ± 11.4 years for control subjects and 40.2 ± 13.1 years for TMJ OA patients; with 4 control and 4 TMJ OA male subjects. The majority of female subjects than male subjects corroborates the sex distribution reported in the literature (22, 23). This study data included 3 sources of diagnostic features: clinical, biomolecular (levels of proteins in serum and saliva), and imaging features.

### Clinical signs and symptoms

The same investigator collected and measured the clinical signs and symptoms of the participants based on the DC/TMD criteria (1, 2). The variables measured and selected for further statistical analysis were: Age, Pain began in years - TMJ OA group only, Current Facial Pain -TMJ OA group only, Worst Facial Pain in last 6 months -TMJ OA group only, Average Pain -TMJ OA group only, Last 6 Months Distressed by Headaches, Last 6 Months Distressed by Muscle Soreness, Vertical Range Unassisted Mouth Opening Without Pain (mm), Vertical Range Unassisted Maximum (mm), Vertical Range Assisted Maximum (mm).

### Biomolecular data

The participants had 5 ml of venous blood collected by a trained nurse at the University of Michigan. The blood was centrifuged for 20 min at 1,000 RPM to separate only the serum that was then aliquoted in 2 ml Eppendorf tubes and stored at −8 °C. For the saliva collection, the participants received a 14 ml sterile test tube with a funnel inserted; they were instructed to tilt their head forward and drip the saliva off into the tube until 2 ml was collected. They were informed to not spit, talk, or swallow during this process. We evaluated 14 proteins (10) in serum and saliva associated with nociception, inflammation, angiogenesis and bone resorption: 6ckine, Angiogenin, BDNF, CXCL16, ENA-78, MMP-3, MMP-7, OPG, PAI-1, TGFb1, TIMP-1, TRANCE, VE-Cadherin and VEGF. However, the expression of 6ckine was below the limit of detection in the serum and saliva samples in this study, and MMP-3 was not expressed in saliva. Those proteins were selected in a previous study that detected these markers in the TMJ synovial fluid and saliva of OA patients, showing correlations with bone surface changes (24). Custom human quantibody protein microarrays obtained from RayBiotech, Inc. Norcross, GA, was used to quantitatively assess the saliva and serum samples for the 14 specific biomarkers. Each participant had duplicates run for the saliva and serum samples.

### Imaging

**FIGURE 1:**
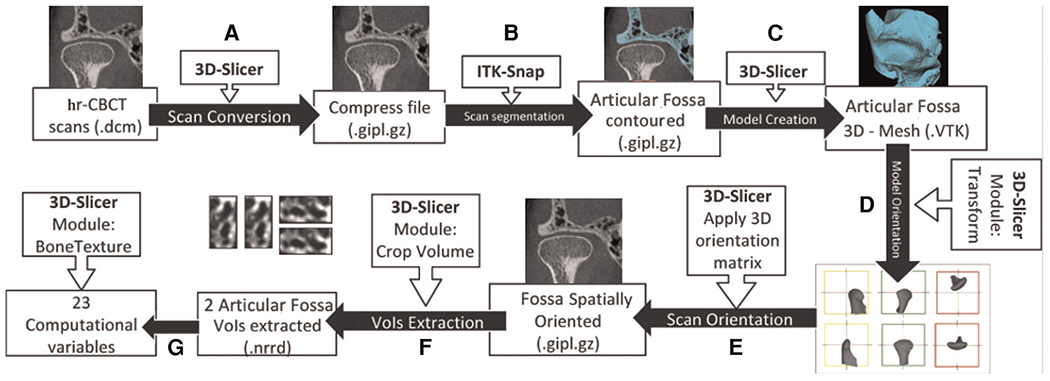
Image processing workflow adapted from bianchi et al. (2021) using 3D Slicer and ITK-SNAP open source software. (**A**) hr-CBCT files were anonymized and compressed. (**B**) The condyle and articular fossa were segmented. (**C**) 3D Slicer was used to convert the segmented articular fossa volume to a 3D surface. (**D**) Using the “transform” module, a standardized spatial orientation for each 3D TMJ bones model was made. Left TMJ scans were mirrored to the right side. (**E**) The spatial orientation matrix created in the last step was applied to the TMJ scan. (**F**) Using the “crop-volume” tool, two regions of the articular fossa (anterolateral, and articular eminence) were selected. (**G**) Using the “BoneTexture” module, all of the radiomic variables were computed.

**FIGURE 2:**
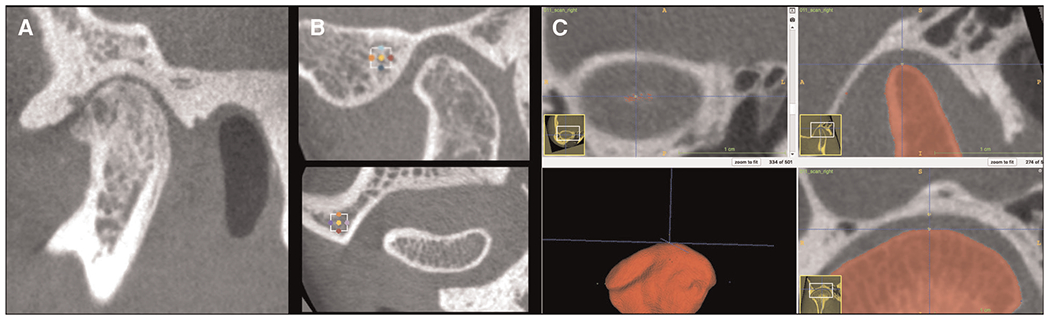
TMJ imaging protocol. (**A**) Small FOV hr-CBCT scans for TMJ imaging features analysis. Note that marked bone destruction is seen in the condyle and the articular fossa also shows erosion. (**B**) Volumes of interest (VOIs) in the lateral portion of the articular fossa and in the articular eminence regions. (**C**) Superior condylar-to-fossa distance as indicated by the blue axis line seen in the coronal and sagittal views.

All small field of view 0.08 mm isotropic voxel CBCT scans were acquired using a 3D Accuitomo scanner (J. Morita Mfg. Corp., Tokyo, Japan). The TMJ acquisition protocol was as follows: field of view (FOV) of 40 × 40 mm, 90 kVp, 5 mAs and scanning time of 30.8s. The limitation of the exposure to the smallest FOV possible is in accordance to the ALARA (as low as reasonably achievable) principle, and this radiation reduction to the patient, maintaining or even improving the level of precision and accuracy in the diagnosis, supports the concept “as low as diagnostically acceptable” (ALADA) (25). Imaging features of one condyle per patient were included to reduce possible bias due to non-specific side data in systemic biological samples and comorbidities, technical problems in the hr-CBCT image acquisition, and presence of unilateral TMJ OA. The detailed image analysis protocol provided applied to the articular fossa region is shown in Figure 1 and all imaging features included have previously been validated for the mandibular condyle by Bianchi, et al (2021) (19) using 3D Slicer (26) and ITK-SNAP (27) open-source software. Anterolateral and articular eminence volume of interest (VOI) articular fossa regions in the FOV (Figure 2A) were selected and extracted using the “crop-volume” module in 3D Slicer (Figure 2B) with 30 × 30 × 30 slices. The posterior regions of the articular fossa were not included due to the presence of air cells in the temporal bone samples and difficulty distinguishing from trabecular bone. A total of 23 surrogate imaging biomarkers were evaluated (19, 28–30), as described in the Table 1. The BoneTexture module in 3D Slicer was used to compute the bone imaging biomarkers and obtain the subchondral bone microstructure values. The software computation parameters were chosen based on the pilot calibration studies from Bianchi et al (2021). The following computational software parameters were selected: (1) for GLCM: mask “inside” value = 1; number of bins = 10; voxel intensity range min = −1,000, max = 2,500; neighborhood radius = 4; (2) for GLRLM: mask “inside” value = 1; number of bins = 10; voxel intensity range min = −1,000, max = 2,500; distance range min = 0, max = 1; neighborhood radius = 4. For bone morphometry (BM), the software parameters were threshold = 250 and neighborhood radius = 4. Five measurements of joint space (Figure 2C) were measured as condylar-to-fossa distances (anterior, anterolateral, medial, superior and posterior). The statistical analysis of the imaging protocol was performed using IBM SPSS Statistics version 27.0 (IBM Corp., Armonk, NY). With an interval of 2 weeks between repeated measures, intra-class correlation coefficients (ICC) were used to assess the study error of the method in the selection of VOIs and computation of radiomic and bone morphometry features, as well as repeatability and interobserver reproducibility of joint space measures. The t-test for independent samples was used to compare the TMJ OA and Control groups with Levene’s Test for Equality of Variances to determine the assumption for homogeneity of variance.

### Diagnostic performance of the markers in machine learning algorithms

The data from this study was incorporated into two artificial intelligence-based tools – TMJOAI (TMJ Osteoarthritis Artificial Intelligence) tool (31) that integrates biological, clinical and imaging data; and the TMJPI (TMJ Privileged Information) tool (32). The Learning Using Privileged Information (LUPI) implemented in the TMJPI tool uses biological data to train the machine learning model but classifies new patients based on clinical and imaging data only, which is the current standard of care. These tools are available in an open-source web system DSCI (Data Storage for Computation and Integration) used for data management with storage and integration of patient information from multiple sources (33).

The TMJOAI tool approach included feature normalization, selection, and model evaluation. We normalized all features to have zero mean and one standard deviation. Next, we calculated the AUC (Area Under the Curve), *p*-value and q-value from a two-sample Mann-Whitney U test to evaluate the significance of each feature. Then, we performed cross-validation (CV) to avoid overfitting – 100 times five-fold CV – resulting in 500 models in total. Each subject was predicted by the ensemble (averaging) of 100 models whose training set did not include that subject. Top main effect features and interactions, filtered with AUC > 0.7 and AUC > 0.65, respectively, calculated from the training subjects were then fed into models to make diagnostic predictions. We trained Extreme Gradient Boosting (XGBoost) (34) and Light Gradient Boosting Machine (LightGBM) (35) machine learning models. For both XGBoost and LightGBM models, we fixed the depth D = 1, and tuned the iteration steps by further splitting the training subjects into training and validation subjects. The following metrics were calculated to evaluate the performances of the model: accuracy, precision, recall, F1-score, and AUC, where AUC was chosen as the evaluation criterion to measure the test’s discriminative ability, i.e., how good is the test in a given clinical situation, with an AUC > 0.7–0.8 as fair, 0.81–0.9 as good and 0.91–1 as very good (36).

The TMJPI tool approach tested the performance of RVFL and KRVFL+ models using biological data as privileged information (32). Considering that biological data is not routinely acquired for TMJ OA patients, we performed five-fold cross-validation and hyper-parameter tuning using a grid-search approach, utilized feature selection approaches such as normalized mutual information feature selection (NMIFS), MRMR (maximum relevancy minimum redundancy) and calculated Shapley Additive explanations values to rank features by their importance (37). We tested the performance of the TMJPI model using AUC, F1-score, sensitivity, specificity, precision, accuracy.

## Results

In the articular eminence and anterolateral VOIs, 22 of the 23 proposed markers had an ICC value of greater than 0.8, indicating good repeatability of these values. In the articular eminence, the ICC value for Cluster Shade was 0.549, and in the anterolateral region, the ICC value for Correlation was 0.539, and these values were excluded from the machine learning models. The ICC values for all five distances in the 3D measurement were greater than 0.8, indicating good repeatability and reproducibility. Statistical significance was detected between patients exhibiting early to moderate stages of TMJ OA and control patients in the 3D measurement of the superior condyle-to-fossa distance (*p* = 0.013) with diseased patients exhibiting a smaller superior condyle-to-fossa distance.

Using the TMJOAI tool, we found that articular fossa radiomics, bone morphometry and joint space data improved the performance of machine learning models in detecting TMJ OA status mainly through interaction effects among the integrated features. The best performing machine learning model was LightGBM model, even better than XGBoost + LightGBM combined, with the highest AUCs and F1- scores. Our results in Table 2 show that the LightGBM model now implemented in the TMJOAI with these features and interactions achieves the accuracy of 0.804, AUC 0.842, and F1-score 0.804 to diagnose the TMJ OA status with 3,081 features interactions.

**FIGURE 3:**
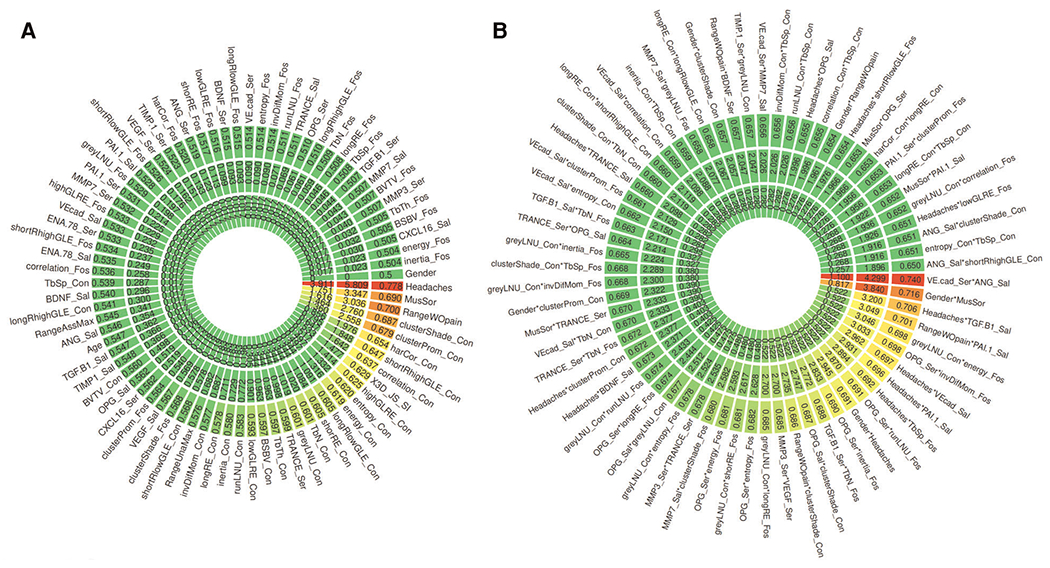
General association analysis of risk factors for 79 features (**A**) and 66 top interactions (**B**). (**A,B**) The outer circle shows the AUC, middle circle shows the *p*-values, and the inner circle shows the q-values for each single feature.

**FIGURE 4:**
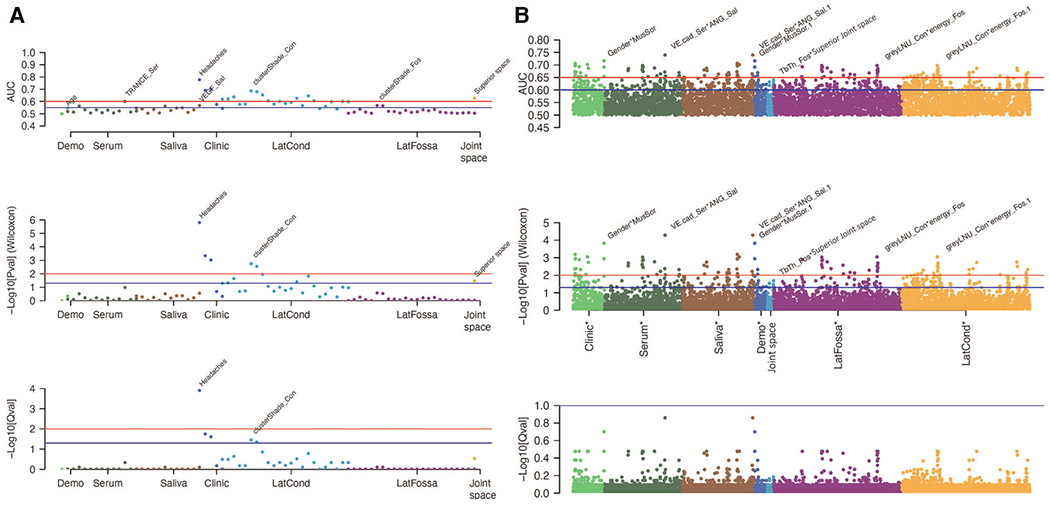
Graphic displays of 79 features (**A**) and 3,081 interactions (**B**). (**A,B**) The upper graphic shows the AUC, the middle graph shows the *p*-values, and the lower category shows the *q*-values for each category of features.

**FIGURE 5:**
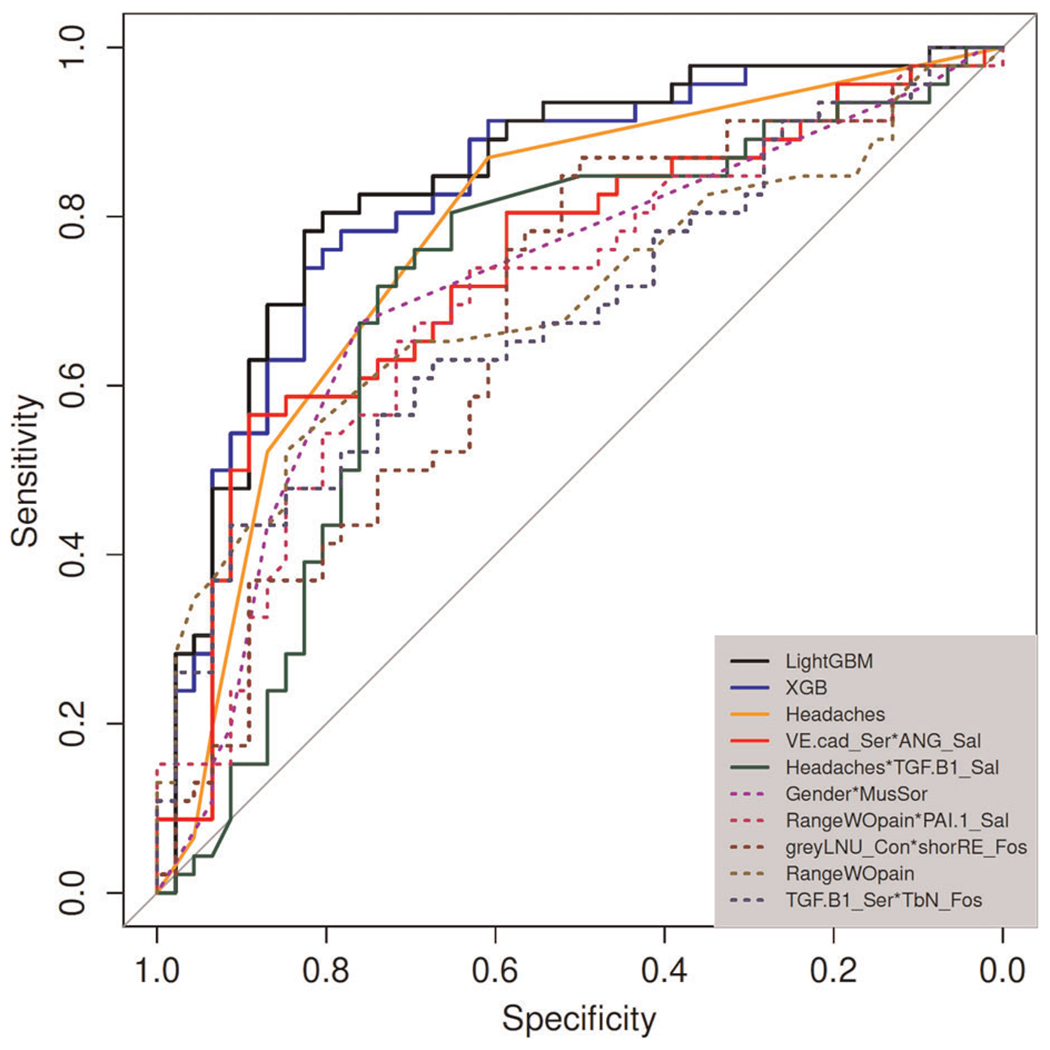
ROC curves of diagnostic sensitivity and specificity for individual features with top mean importance and the mean prediction of XGBoost and LightGBM.

**FIGURE 6:**
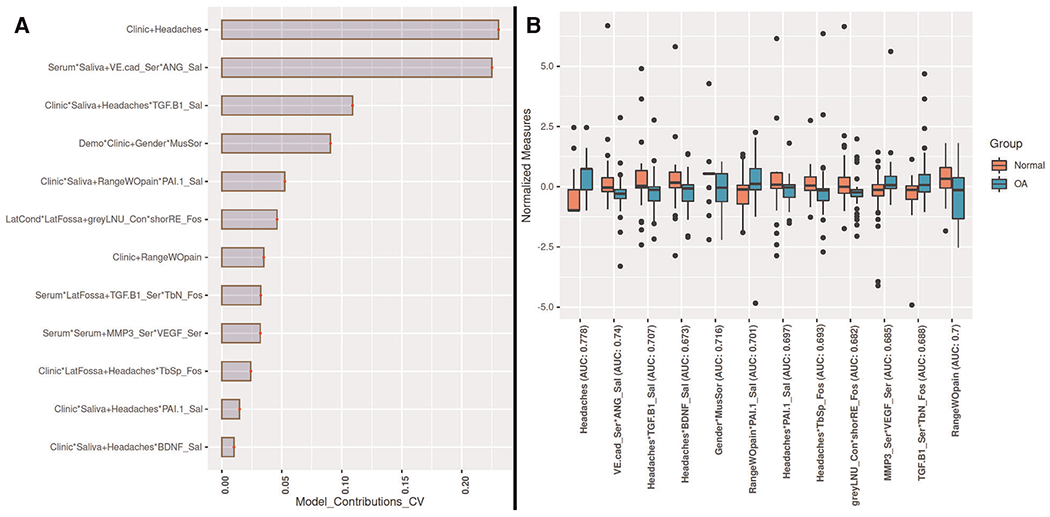
Top 12 features in the LightGBM prediction model. (**A**) Mean contribution (according to feature importance) greater than 90% for 100 times 5-fold CV. (**B**) Boxplots of normalized features to diagnose disease status.

The values for the AUC, *p*-value, and *q*-value for all features are shown in Figures 3A, 4A. Figure 3A shows the AUC (upper plot), *p*-value (middle plot) and *q*-value (lower plot) for each category of variables (biological, clinical, condylar radiomics, articular fossa radiomics, and joint space). Figure 5 shows the 12 features with >90% top contributions sum: Headaches, VE-cadherin in Serum and Angiogenin in Saliva, TGF-*β*1 in Saliva and Headaches, Gender and Muscle Soreness, PA1 in Saliva and Range of mouth opening without pain, Lateral Condyle Grey Level Non-Uniformity and Lateral Fossa Short Run Emphasis, Range of mouth opening without pain, TGF-*β*1 in Serum and Lateral Fossa Trabeculae number, MMP3 in Serum and VEGF in Serum, Headaches and Lateral Fossa Trabecular spacing, Headaches and PA1 in Saliva, and Headaches and BDNF in Saliva. Most of the features with significant AUC values are clinical or condylar radiomics; no fossa radiomic or joint space features are detected with AUC > 0.65 (Figure 3A). The highest AUC value for a main effect fossa radiomic or joint space features was the superior joint space distance (Figure 3A); the interaction of Headaches and Lateral Fossa Trabecular spacing, Lateral Condyle Grey Level Non-Uniformity and Lateral Fossa Short Run Emphasis, and TGF-*β*1 in Serum and Lateral Fossa Trabeculae number were found to significantly contribute to the prediction of TMJ OA status (Figures 3B, 4B, 6A).

For articular fossa markers, prediction models show that the interaction between Lateral Condyle Grey Level Non-Uniformity and Lateral Fossa Short Run Emphasis, TGF-*β*1 in Serum and Lateral Fossa Trabeculae number, and Headaches and Lateral Fossa Trabecular spacing, are found to be top features for accurate diagnosis of early stages of this clinical condition (Figure 6A). After the selection of the best features and interactions (Figure 6A), Figure 6B displays the boxplots for comparison between OA and control groups with corresponding AUCs, further demonstrating performance in diagnosis of TMJ OA status. Figure 5B shows the ROC curves of diagnostic sensitivity and specificity for individual features with top mean importance and the mean prediction of XGBoost, LightGBM and their ensemble with LightGBM demonstrating the largest ROC curve and highest discriminative ability of the models and features (Table 2).

Using TMJPI, when combining clinical, radiomic (condyle and fossa), and 3D joint space features, LUPI-based models with additional biological features significantly enhanced the model performance on clinical, joint space measurement, and condyle datasets. The best clinical performance was obtained with the KRVFL+ model, keeping all clinical criteria and applying feature selection on the condyle and joint space features (Evaluation metrics shown in Table 2). The Shapley ranking of features based on their importance indicated 12 top features: 3 Clinical (Headaches, Muscle Soreness, Vertical Range Unassisted Mouth Opening Without Pain), 7 condylar radiomics and morphometry (Trabecular thickness, ShortRunHighGreyLevelEmphasis, Cluster Prominence, Entropy, Correlation, InverseDifferenceMoment and Energy) and 2 joint space features (Superior and Medial).

## Discussion

This study demonstrates the diagnostic performance of joint space distances and radiomic biomarkers of the subchondral bone in hr-CBCT scans of TMJ OA patients in the articular fossa region. Surrogate articular fossa bone morphometry and textural features were not significantly different between TMJ OA patients and controls, whereas the superior joint space was significantly smaller in TMJ OA patients. This may suggest that joint space narrowing in the superior region may serve as an early sign of TMJ OA as found in previous studies (22).

The inclusion of quantitative articular fossa radiomics and joint space to machine-learning algorithms proved to be useful in enhancing the performance of TMJ OA classifiers. While articular fossa imaging biomarkers alone may not be diagnostic of early disease stages, through interactions with condylar, clinical and biological changes, fossa features may serve to strengthen the performance of machine-learning algorithms. Headaches and Range of mouth opening without pain and interactions of VE-cadherin in Serum and Angiogenin in Saliva, TGF-*β*1 in Saliva and Headaches, Gender and Muscle Soreness; PA1 in Saliva and Range of mouth opening without pain, Lateral Condyle Grey Level Non-Uniformity and Lateral Fossa Short Run Emphasis, TGF-*β*1 in Serum and Lateral Fossa Trabeculae number, MMP3 in Serum and VEGF in Serum, Headaches and Lateral Fossa Trabecular spacing, Headaches and PA1 in Saliva, and Headaches and BDNF in Saliva were the top features/interactions to accurately diagnose early stages of this clinical condition. Three of these interactions include fossa components showing that the assessment of fossa markers proves useful in diagnosis, as shown in our results with TMJOAI interaction effects. Therefore, while the articular fossa markers alone are not ranked among the features with highest AUC (Figures 3A, 4A), many articular fossa feature interactions present higher AUC (Figures 3B, 4B). The prediction model shows that the interaction between Lateral Condyle Grey Level Non-Uniformity and Lateral Fossa Short Run Emphasis, TGF-*β*1 in Serum and Lateral Fossa Trabeculae number, Headaches and Lateral Fossa Trabecular spacing are found to be top contributing features for accurate diagnosis of early stages of this clinical condition (Figure 6). This finding is similar to that of Bianchi et al. (2020) (10) prediction models that showed that the interaction between VE-cadherin in Serum and Angiogenin in Saliva, Headaches and PA1 in Saliva, TGF-*β*1 in Saliva and Headaches, VE-cad_Sal*Headaches (AUC= 0.698), TGF-*β*1 in Saliva and Headaches, and PA1 in Saliva and Range of mouth opening without pain are top features with mean >80% contribution to the information gain in the XGBoost and LightGBM predictive models. Therefore, our preliminary results suggest that while the condyle imaging features may be more important in regard to main effect (Figure 3A), the fossa features may have a larger contributing factor in terms of interaction effects (Figure 3B) – though future studies with larger sample sizes are needed.

In TMJPI, the machine learning models only tested the original features and the main effect of each feature in overall TMJ OA status, whereas in TMJOAI, the machine models also tested interactions between features. This could explain why model performance decreased in TMJPI with the inclusion of all radiomic features, as TMJOAI models showed that the radiomic contribution is predominantly through interaction effects. Currently, the TMJPI tool is limited in computational approaches for testing features interactions due to the fact that it is supercomputing intensive and it takes a long time to train the model with interactions built in. As our baseline sample recruitment continues, larger sample sizes will allow further training of non-LUPI and LUPI-based algorithms on the TMJ OA datasets using grid search and 5-fold cross-validation (CV) on the training set to determine the optimal hyperparameters and features for each algorithm.

A limitation of the study similar to that of Bianchi et al. (2021) (10) was the use of the DC/TMD (1, 2) imaging criteria to confirm the diagnosis of the TMJ OA; however, the hr-CBCT used has a voxel size of 0.08 mm^3^, showing higher resolution and details than described in the DC/TMD imaging data, which uses CT scans with 0.7–1 mm slice thickness. Even with the addition of radiographic criteria to the DC/TMD – the standardized and widely used protocols for TMJ OA assessment – there is still a reliance on subjective radiological interpretation of pre-existing bone changes and clinical symptoms (1, 2). Furthermore, the cross-sectional study design does not allow assessment of the disease progression and how different disease stages affect the proposed biomarkers. This study was conducted only at baseline – providing another classification of disease vs. control that is already available with imaging and clinical symptoms. However, the ultimate goal of this work is the longitudinal assessments that will follow that test the potential of these baseline predictor values to also be predictive of risk of disease progression. This is valuable in determining which subjects are at greater risk of worsening over time, or which subjects would respond better to conservative approaches such as a mouthguard or splint therapy. Therefore, these initial markers detected in this study can serve as surrogate markers to be tested in future studies of risk of disease progression. Future studies using the proposed machine learning models and longitudinal data will provide better information on the feature’s behavior and disease progression. However, a drawback currently is that feature extraction from Cone-Beam Computed Tomography (CBCT) images remains time consuming before this integrative model can be applied in larger scale studies. Automatization of image processing steps and further refinements in machine-learning algorithms to detect early markers of disease have the potential to improve prediction of disease progression and severity to ultimately better serve and treat patients with TMJ OA.

## Conclusion

Our results indicate that the condyle imaging features may be more important in regard to main effect; whereas, for interaction effect, the fossa features may play a crucial role in the diagnosis of TMJ OA. Narrowing of the superior joint space was observed in TMJ OA patients. We developed a methodology for extraction of articular fossa radiomics and joint space distances utilizing machine learning for a comprehensive integration and management of data from various sources to improve articular joint health and predict patient-specific TMJ OA status.

## Figures and Tables

**TABLE 1:** Description of the variables for radiomic and bone morphometry features.

Features	Variables	Definitions
Grey-Level Co-occurrence Matrix (GLCM)	Energy	Uniformity of the grey-level textural organization.
	Entropy	Randomization of the grey-level distribution.
	Correlation	Grey-level linear dependence among the pixels.
	Inverse Difference Moment	Local homogeneity of the grey-level distribution.
	Inertia	Contrast between a pixel and its neighbor.
	Cluster Shade	Skewness and uniformity of the grey-level distribution.
	Cluster Prominence	Skewness and asymmetry of the grey-level distribution.
	Haralick Correlation	Linear dependence between the pixels.
Grey-Level Run Length Matrix (GLRLM)	Short Run Emphasis	Distribution of short run lengths.
	Long Run Emphasis	Distribution of long run lengths.
	Grey Level Non Uniformity	Variability of the grey-level intensity.
	Run Length Non Uniformity	Similarity of run lengths in the image.
	Low Grey Level Run Emphasis	Distribution of the lower grey-level values.
	High Grey Level Run Emphasis	Distribution of the higher grey-level values.
	Short Run Low Grey	Joint distribution of shorter run
	Level Run Emphasis	lengths with lower grey-level values.
	Short Run High Grey	Joint distribution of shorter run
	Level Run Emphasis	lengths with higher grey-level values.
	Long Run Low Grey	Joint distribution of long run
	Level Run Emphasis	lengths with lower grey-level values.
	Long Run High Grey	Joint distribution of long run
	Level Run Emphasis	lengths with higher grey-level values.
Bone Morphometry	BV/TV	Ratio between bone volume and total volume.
	Tb.Th	Trabecular thickness.
	Tb.Sp	Trabecular separation.
	Tb.N	Trabecular number.
	BS/BV	Ratio between bone surface and bone volume.

**TABLE 2:** Comparison metrics for the final model in TMJOAI and TMJPI. Multi-class precision and recall for the TMJOAI models are shown as Precision1 and Recall1 for OA and Precision0 and Recall0 for control groups, respectively, and the final F1-score was calculated by taking the macro average of the two classes’ F-1 scores (38, 39).

TMJOAI Models	AUC	Accuracy	Precision1	Precision0	Recall1	Recall0	F1 Score
XGBoost	0.829	0.772	0.778	0.766	0.761	0.783	0.772
LightGBM	0.842	0.804	0.804	0.804	0.804	0.804	0.804
XGBoost + LightGBM	0.837	0.783	0.783	0.783	0.783	0.783	0.783
TMJPI Model	AUC	Accuracy	Precision	Sensitivity	Specificity	F1 score	
KRVFL+	0.809	0.709	0.774	0.627	0.791	0.661	

## Data Availability

The raw data supporting the conclusions of this article will be made available by the authors, without undue reservation.
